# Dental Age in Orthodontic Patients with Different Skeletal Patterns

**DOI:** 10.1155/2017/8976284

**Published:** 2017-03-16

**Authors:** Tomislav Lauc, Enita Nakaš, Melina Latić-Dautović, Vildana Džemidžić, Alisa Tiro, Ivana Rupić, Mirjana Kostić, Ivan Galić

**Affiliations:** ^1^Study of Anthropology, Faculty of Social Sciences and Humanities, University of Zagreb, 10000 Zagreb, Croatia; ^2^Department of Dental Medicine, Faculty of Medicine, University of Osijek, 31000 Osijek, Croatia; ^3^Department of Orthodontics, School of Dental Medicine, University of Sarajevo, 71000 Sarajevo, Bosnia and Herzegovina; ^4^Dental Clinic Apolonija, 10000 Zagreb, Croatia; ^5^Dental Department, The Public Institution Health Centre of Sarajevo Canton, 71000 Sarajevo, Bosnia and Herzegovina; ^6^University of Zagreb, School of Medicine, Croatian Health Insurance Fund, 10000 Zagreb, Croatia; ^7^School of Medicine, University of Split, 21000 Split, Croatia

## Abstract

*Objective*. To evaluate the difference between chronological and dental age, calculated by Willems and Cameriere methods, in various skeletal patterns according to Steiner's ANB Classification.* Methods*. This retrospective cross-sectional study comprised the sample of 776 participants aged between 7 and 15 years (368 males and 408 females). For each participant, panoramic images (OPT) and laterolateral cephalograms (LC) were collected from the medical database. On LC ANB angle was measured; on OPT dental age (DA) was calculated while chronological age (CA) and sex were recorded. The sample was divided into three subgroups (Class I, Class II, and Class III) with similar distribution based on the chronological age and ANB angle. CA was calculated as the difference between the date of OPT imaging and the date of birth, while DA was evaluated using Willems and Cameriere methods. ANB angle was measured on LC by two independent investigators using the cephalometric software. Differences between sexes and the difference between dental and chronological age were tested by independent and paired samples *t*-test, respectively; one-way ANOVA was used to test differences among ANB classes with Tukey post hoc test to compare specific pairs of ANB classes.* Results*. The significant difference was found between Class III and other two skeletal classes in males using both dental age estimation methods. In Class III males dental age was ahead averagely by 0.41 years when using Willems method, while Cameriere method overestimated CA for 0.22 years.* Conclusion*. In males with Class III skeletal pattern, dental development is faster than in Classes I and II skeletal pattern. This faster development is not present in females.

## 1. Introduction

Dental development is a multilevel process, and it entails molecular and cellular interactions, which have macroscopic and clinical phenotypic outcomes. The process of dental development is multidimensional, requiring developments in the three spatial dimensions with the fourth dimension of time. It is progressive, occurring over an extended period, yet at critical stages of development [1, 2]. In the same time of intensive changes, growth and development of different bones constituting the facial skeleton do not exhibit the same rate of growth [3]. As the teeth grow in the bone substratum, under the similar growth factors, it can be expected that the growth factors can have similar influence onto dental and bone growth intensity in the same jaws.

It is well known that the growth is an important aspect in dentofacial orthopedics, as treatment outcomes and stability may be influenced by the maturational status of the patient [4]. Correlation and possible Influence of facial pattern of the growth and dental development have been intensively studied earlier [5–9]. All previous studies investigated the correlation between vertical growth pattern and dental development. At the same time, there is a limited amount of research that investigated horizontal skeletal growth pattern and dental development; even some studies showed that the rate of growth is different depending on the pattern of the sagittal skeletal growth [10, 11].

Many biological indicators can be used for determination of the growth and development such as body weight, body height, dental development, or skeletal development. The X-ray images are recognized as a reliable method for the exact determination of skeletal pattern, as well as for the dental development stage. Sagittal skeletal relationships can be determined from LC, with widely used Steiner's [12] sagittal analysis where the analysis of ANB angle indicates the magnitude of skeletal jaw discrepancy [13–15].

Different age estimation methods on developing teeth were presented over last 70 years [16, 17]. Most of the methods on developing teeth evaluate mandibular teeth from one side while some of them use all or just specific set of teeth from single or both jaws [18–22]. Demirjian method, scoring system introduced in 1973, is one of the most widely used methods for estimating dental developing stage [23]. It is based on an assessment of mineralization of seven teeth from one side of mandible where development from crypt formation until mature was divided into eight stages, marked with alphabet letters from A to H [19]. This method was used in many populations, including studies in Bosnia and Herzegovina [24, 25]. A meta-analysis by Yan et al. [26], based on 26 studies, showed that Demirjian's method overestimated dental age by 4.2 months in males and 4.68 months in females. Comparative studies of different dental methods have shown that another Willems method exhibited smaller error rate when compared to the real age [16, 20, 27, 28]. The other recent method developed by Cameriere et al. [29] introduced a different approach on the same set of seven teeth, analyzing a teeth maturation as the proportion of open apices and heights of the roots. Additional variables in the regression model were sex, the number of teeth with closed apices, and the sum of the proportion of all teeth in development while ethnicity was not a significant factor [29]. Willems and Cameriere's methods were found to be reliable and accurate in many populations and also confirmed as the appropriate method for evaluating dental development stage in Bosnia and Herzegovina population [16].

Most of the previous studies estimated dental age in general population without taking account of the possible effect of skeletal pattern on the dental development stage [16, 25, 30, 31]. However, one study by Celikoglu et al. [30] evaluated Demirjian dental age in patients with and without skeletal malocclusions. This study showed that girls with skeletal Class III according to the ANB angle classification by Steiner (ANB) have significantly earlier dental development than other Class I or Class II participants in the study [12]. Their result is in concordance with our hypothesis that the increase in skeletal growth, as the consequence of growth factors in the bone can influence the increase of the dental development.

Therefore, the purpose of this study was to investigate if patients with Class II patterns (ANB > 4 degrees) or Class III patterns (ANB 0 degrees or negative) have different timing of dental development. If so, that difference should be taken in calculation when age estimation analyses in dental forensics are provided, or in the planning of functional orthodontic treatment where the skeletal and dental age can be different from the chronological age of the patient.

## 2. Materials and Methods

This is a retrospective cross-sectional study of dental age estimation in orthodontic patients from the University of Sarajevo School of Dental Medicine Orthodontic Department. Ethical approval for the study was obtained from the School of Dental Medicine Ethical Committee, and the study was performed according to World Medical Association Declaration of Helsinki for ethical principles for medical research involving human subjects [32].

The sample consisted of 776 participants aged between 7 and 15 years (368 males and 408 females). The first inclusion criterion for each participant was that the panoramic image (OPT) and lateral cephalogram (LC) from the medical records were gathered at the same time, before any orthodontic treatment. The sample was divided into three subgroups (Stainer's skeletal Class I, Class II, and Class III according to ANB angle) with the similar distribution based on the chronological age.

All OPT and LC were recorded on the same X-ray scanner (KODAK 8000C Digital Panoramic and Cephalometric System, Carestream, France). Chronological age (CA) was calculated as the difference between the date of OPT scanning and the date of birth from the medical record.

Skeletal class was evaluated on each LC according to Steiner's A point-Nasion-B point angle (ANB angle) [12] by two independent investigators. No interexaminer difference was found for ANB angle calculation. Briefly, for ANB angle, A point presents the most concave point of the anterior maxillar base; Nasion (N) presents the most anterior point of the frontonasal suture, while B point presents the most concave point of the anterior contour of mandibular symphysis. Steiner's classification recognizes different skeletal patterns according to ANB angle, Class I ranges from 0 to 4 degrees, Class II presents angle of over 4 degrees, and Class III is ANB angle of negative value or 0 degrees.

Dental age was calculated according to Willems and Cameriere dental age estimation method, which shows the smallest error of age estimation [16, 27]. Willems' method is based on the assessment of Demirjian stages on seven mandibular teeth [19]. OPTs of French-Canadian children have been evaluated and seven permanent teeth from the left side of the mandible, excluding third molars, have been rated [19]. Demirjian stages are derived from evaluation of eight mineralization stages, alphabetically marked from A to H. The first stage A represents a beginning of calcification, seen at the superior level of the dental crypt, without fusion of this calcification, while the last stage H represents finished calcification of the tooth with apical ends of the roots completely closed [19]. For each stage, Demirjian presented specific self-weighted score and summed score on all seven teeth present a dental maturity score which can be converted to dental age [19]. Willems et al. [33] in 2001 revisited the original Demirjian method in a Belgian population and adopted the original Demirjian's scoring system by using a weighted ANOVA. The ANOVA model was used with all seven teeth as covariates for boys and girls separately. Specific tables for each sex with corresponding age scores expressed directly in years of each stage for each of the seven left mandibular teeth for age calculation were presented [33].

Cameriere's method was based on regression analysis of age as dependent variable and proportions of measurements of open apices and heights of the same seven mandibular teeth on the OPT, where sex (*g*) and number of teeth with finished maturation of root apex (*N*_0_) are important dependent variables in calculating DA [29, 34]. Briefly, all teeth without complete root development or with open apices were examined and the distance (*A*_*i*_, *i* = 1,…, 5) between the inner side of the open apex was measured. For teeth with two roots, (*A*_*i*_, *i* = 6,7), the sum of the distances between the inner sides of the two open apices was calculated. Distances were normalized by dividing by the tooth length (*L*_*i*_, *i* = 1,…, 7) to minimize the effect of differences among X-rays in magnification and angulation [34]. Dental age was calculated according to the European formula: Age = 8.387 + 0.282*g* − 1.692*x*_5_ + 0.835*N*_0_ − 0.116*s* − 0.139*s∗N*_0_, where *g* is a variable, with *g* = 1 for boys and *g* = 0 for girls,* s* is the sum of the normalized open apices of the seven left permanent developing mandibular teeth (*x*_*i*_ = *A*_*i*_/*L*_*i*_,  *i* = 1,…, 7), and *x*_5_ is the normalized measurement of the second premolar [29].

The results were tested for each sex separately. A Shapiro-Wilk test and normal Q-Q Plots showed normal distribution of the differences between estimated and chronological age or residuals for both methods [35]. Differences between dental and chronological age for both methods were evaluated with paired samples *t*-test; one-way ANOVA was used to test the effect of ANB classes on differences between estimated and chronological age, with Tukey as the post hoc test [35]. Cohen Kappa was used to verify intraobserver and interobserver agreement in Demirjian staging and in a number of teeth with closed apices as evaluated by Cameriere's method between the two independent observers, as well as for two measurements by the same observer [36]. Intraclass correlation coefficient (ICC) was used to test calculated a dental age for the intraobserver and interobserver agreements [36]. SPSS Statistics 16.0 for Windows (SPSS Inc., Chicago, IL) was used for statistical analysis, and statistical significance was set at 0.05.

## 3. Results

In all participants involved in this study dental age estimation using both methods and classification into specific ANB angle skeletal class was possible to evaluate. Distribution of sample according to sex, ANB skeletal class, and age was presented in Table 1.

**Figure 1:**
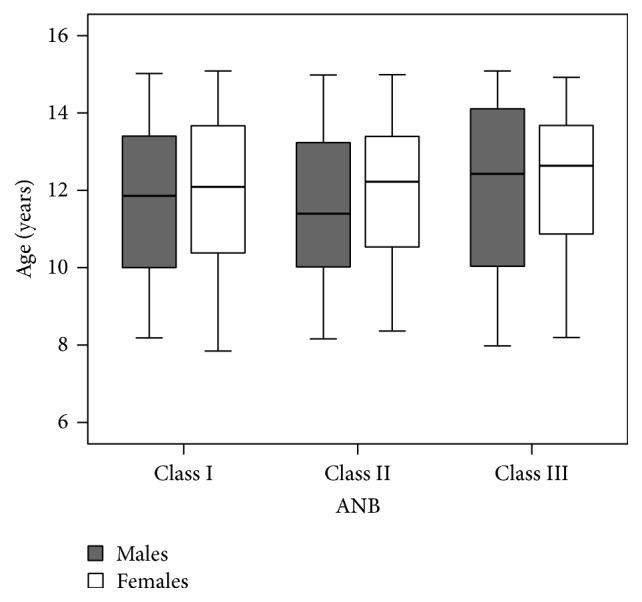
Distribution of the chronological age among ANB skeletal classes.

Cohen Kappa scores, for intraobserver and interobserver agreement between the same and two different observers, were 0.81 (95% CI, 0.72 to 0.90) and 0.72 (95% CI, 0.57 to 0.86), respectively, for scoring Demirjian staging system. Cohen Kappa for scoring the number of teeth with closed apices on Cameriere's method was 1.00. ICC for calculated dental age for the intraobserver and interobserver agreements were 0.98 (95% CI, 0.97 to 0.99) and 0.97 (0.95%, 0.95 to 0.98), respectively, for Willems method and 0.98 (95% CI, 0.97 to 0.99) and 0.97 (95% CI, 0.96 to 0.98), respectively, for Cameriere method. One-way between-groups ANOVA, to test difference of mean chronological ages among different ANB skeletal classes, showed no statistically significant difference in males, *F*(2, 365) = 0.71, *p* = 0.49 and females, *F*(2,405) = 0.54, *p* = 0.58.  Figure 1 shows a finding of the chronological age among ANB skeletal Classes I to III.

**Figure 2:**
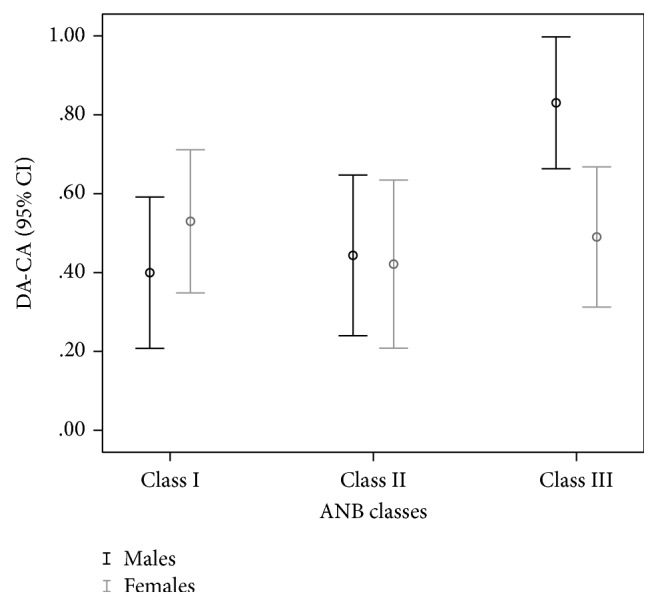
Differences between dental age calculated by the Willems method and chronological age (DA-CA) in years among ANB skeletal classes.

Dental age, calculated by the Willems method, showed a statistically significant overestimation of DA when compared to CA, *p* < 0.001. Average overestimation was 0.57 years with 95% confidence interval (95% CI, 0.46 to 0.68 years) in males and 0.48 years (95% CI, 0.38 to 0.59 years) in females (Table 2). One-way between-groups ANOVA showed statistically significant difference in overestimation among classes in males, *F*(2, 365) = 6.60,  *p* = 0.002, but not in females (Table 3). Post hoc comparison showed that the mean overestimation in males for Class III, 0.83 years (95% CI, 0.66 to 1.00 years), was statistically significantly different from Class I, 0.40 years (95% CI, 0.21 to 0.59 years) (*p* = 0.0008), and Class II, 0.44 years (95% CI, 0.24 to 0.65 years) (*p* = 0.0056). Classes I and II did not differ significantly (Figure 2).

**Figure 3:**
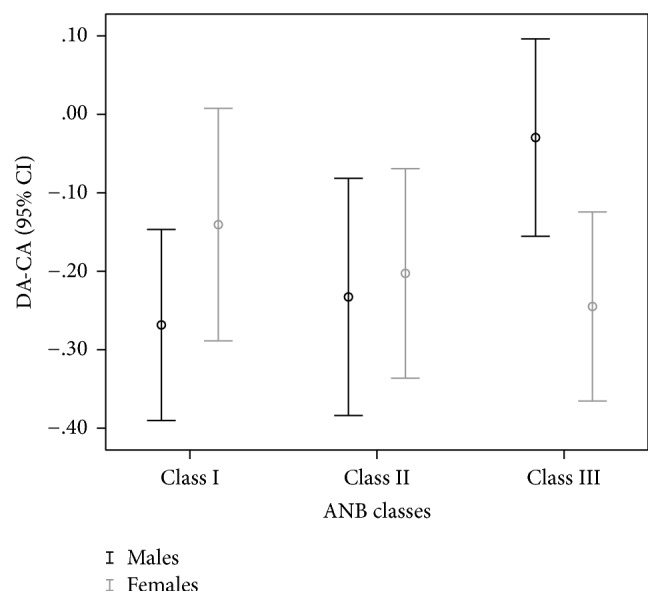
Differences between dental age calculated by the Cameriere method and chronological age (DA-CA) among ANB skeletal classes.

Dental age calculated by the Cameriere method showed an underestimation of CA, which was not statistically significant only in males for Class III, *t* (131) = −0.47, *p* = 0.642 and in females for Class I, *t* (147) = 1.88, *p* = 0.063. Average underestimation was −0.19 years (95% CI, −0.27 to −0.18 years) in males and −0.17 years (95% CI, −0.24 to −0.09 years) in females (Table 2). One-way between-groups ANOVA showed statistically significant difference in underestimation only in males, *F*(2, 365) = 3.99, *p* = 0.019 (Table 3). Post hoc comparison showed that the mean underestimation in males for Class III, −0.02 years (95% CI, −0.16 to 0.10 years), was significantly different from Class I, −0.26 years (95% CI, −0.39 to −0.15 years) (*p* = 0.008), and Class II, −0.23 years (95% CI, −0.38 to −0.08 years) (*p* = 0.037). Classes I and II did not differ significantly, which is presented in Figure 3.

## 4. Discussion

Relevant studies reporting correlations between dental development and skeletal patterns are limited in the recent literature. The influence of skeletal pattern to dental development is still not fully understood, but if different dental development occurs in a various skeletal pattern then the diagnosis of the specific pattern may help to estimate the dental development in forensic dentistry properly and can have the clinical relevance in the planning of orthodontic treatment time.

In this study, we analyzed dental development stage using two dental age evaluation methods and compared the dental development with the pattern of skeletal growth. For the assessment of dental development, we used Willems and Cameriere methods that showed the smallest error between dental and chronological age as published in the recent literature [16]. The sample evaluated in our study was similarly distributed in all ANB classes and across the age range. Upper age of the sample was limited to only those OPTs with evidence of unfinished maturation of the second molars. Older subjects were not qualified for the evaluated methods.

Willems method of dental age evaluation in this sample overestimated the chronological age in all ANB classes and both sexes. This means that the error of Willems method is distributed among the all skeletal patterns and in both sexes. An overestimation was the same among all ANB classes in females, which means that in females dental development is equal among all skeletal patterns. However, in male examinees, dental age in ANB class III was overestimated almost twofold when compared to ANB Class I and/or Class II. This suggests that in Class III males dental development starts earlier than in Class I and/or Class II.

Cameriere method showed a smaller error in the estimation of chronological age when compared to Willems method, and that was negative, which means that Cameriere method of dental age evaluation underestimates chronological age. The mean underestimation was −0.19 years for males and −0.17 years for females. In males, ANB Class III was statistically different when compared to Class I or Class II. Cameriere method underestimated chronological age using evaluation of dental age in males with ANB Class III for only 0.02 years. These findings are in concordance with the explanations using Willems method and also suggest that in males with Class III ANB angle dental development starts earlier than in other skeletal patterns.

Previous study by Celikoglu et al. [30], who used Demirjian method for age estimation, showed that ANB Classes II and III patients were dentally advanced compared to Class I. Principally, they showed that the difference was the highest for their patients with mandibular prognathism or Class III for both sexes, which was statistically significant only in females. Differences in patterns between sexes in our study and study by Celikoglu et al. [30] indicate sex differences using different age estimation method, but the pattern of Class III earlier dental development is consistent between samples.

A similar pattern in the difference of the dental age in males with skeletal Class III or advanced dental maturation, when compared to other two classes, indicates a possible association between this skeletal anomaly and advanced dental maturation. Except for one study [30], there are no investigations that evaluate dental age evaluation in specific malocclusion groups. Jamroz et al. [8] demonstrated that subjects with short anterior facial height presented a slight tendency toward a more advanced dental age than those with long anterior facial height. Uysal et al. [37] found the difference in dental age between examinees with posterior cross-bite and control groups, where subjects with a posterior cross bite had a tendency for a prolonged dental maturation compared to the control individuals with the clinical relevance. No significant side differences in either group were detected.

It is important to stress that the dental age evaluation was calculated according to methods that use lower mandibular teeth from the left side of the mandible. If the mandibular growth is accelerated or started earlier, as it is usual in most Class III, we can expect that growth factors in mandible also influence the dental development of mandibular teeth, as they are only analysed in dental age evaluation methods. If this is the explanation of the difference in dental age in different skeletal pattern, we have to evaluate carefully different skeletal patterns with other age estimation methods in order to give the exact answer: does the skeletal pattern influence the dental development or are the dental age estimation methods dependable of the intensity of growth in the jaw where the teeth for estimation method are located?

## 5. Conclusions

Dental age calculated by Willems method overestimated, while by Cameriere method underestimated the chronological age in all ANB Classes. Both age estimation methods showed the same pattern in males with ANB Class III when compared to other two classes. Dental development in males with Class III was ahead by 0.4 years for Willems method and by 0.2 years for Cameriere method. The results of this investigation suggest that diversity of the skeletal pattern could be connected with the different time of dental development. If so, this should be involved in age estimation methods in dental forensics with the involving skeletal pattern in the process of age estimation or, in orthodontic clinical practice, to have in mind that the intensifying of skeletal growth can increase the dental development in surrounding jaw.

## Figures and Tables

**Table 1:** Distribution of sample according to sex, Steiner's skeletal classes of ANB angle and age.

Age	ANB angle Class I		ANB angle Class II		ANB angle Class III	
	M	F	M	F	M	F
7		4			2	
8	12	8	9	9	18	14
9	22	16	16	16	10	14
10	22	22	16	10	14	8
11	12	18	15	17	14	12
12	22	26	13	26	18	32
13	28	26	16	23	18	34
14	16	24	15	17	34	28
15	2	4			4	

Total	136	148	100	118	132	142

M = males; F = females.

**Table 2:** Comparison of chronological age and dental age calculated by Willems and Cameriere methods in different ANB skeletal classes.

Method	Sex	Class	*N*	Chronological age (CA)		Dental age (DA)		DA-CA		Paired-samples *t*-test	
				Mean	SD	Mean	SD	Mean	SD	*t*(df)	*p*
Willems	Males	I	136	11.71	1.94	12.11	2.54	0.40	1.13	4.1 (135)	<0.001
		II	100	11.67	2.00	12.11	2.54	0.44	1.03	4.3 (99)	<0.001
		III	132	11.96	2.17	12.79	2.65	0.83	0.97	9.8 (131)	<0.001
		Total	368	11.79	2.04	12.36	2.65	0.57	1.06	10.2 (367)	<0.001
	Females	I	148	12.00	2.01	12.52	2.50	0.53	1.12	5.8 (147)	<0.001
		II	118	11.96	1.86	12.38	2.53	0.43	1.17	3.9 (117)	<0.001
		III	142	12.19	1.96	12.68	2.57	0.49	1.07	5.5 (141)	<0.001
		Total	408	12.05	1.95	12.54	2.53	0.48	1.12	8.8 (407)	<0.001

Cameriere	Males	I	136	11.71	1.94	11.44	2.04	−0.26	0.72	−4.36 (135)	<0.001
		II	100	11.67	2.00	11.44	1.90	−0.23	0.76	−3.06 (99)	0.003
		III	132	11.96	2.17	11.93	1.99	−0.02	0.73	−0.47 (131)	0.642
		Total	368	11.79	2.04	11.62	2.00	−0.19	0.80	−4.48 (367)	<0.001
	Females	I	148	12.00	2.01	11.86	1.70	−0.14	0.91	−1.88 (147)	0.063
		II	118	11.96	1.86	11.75	1.72	−0.20	0.73	−3.01 (117)	0.003
		III	142	12.19	1.96	11.94	1.74	−0.24	0.73	−4.02 (141)	<0.001
		Total	408	12.05	1.95	11.86	1.72	−0.17	0.74	−4.92 (407)	<0.001

**Table 3:** Summary ANOVA tables to test the differences in DA-CA among ANB skeletal classes for Willems and Cameriere methods.

Method	Sex		Sum of squares	df	Mean square	*F*	*p*
Willems	Males	Between groups	14.49	2	7.24	6.60	0.002
		Within groups	400.67	365	1.10		
		Total	415.16	367			
	Females	Between groups	0.78	2	0.39	0.31	0.732
		Within groups	505.26	405	1.25		
		Total	506.04	407			

Cameriere	Males	Between groups	4.31	2	2.15	3.99	0.019
		Within groups	196.84	365	0.54		
		Total	201.14	367			
	Females	Between groups	0.80	2	0.40	0.62	0.536
		Within groups	259.14	405	0.64		
		Total	259.93	407			
